# Square-Stepping Exercise Program Effects on Fall-Related Fitness and BDNF Levels in Older Adults in Korea: A Randomized Controlled Trial

**DOI:** 10.3390/ijerph19127033

**Published:** 2022-06-08

**Authors:** Hyo-Jeong Cha, Kwi-Baek Kim, Seung-Yup Baek

**Affiliations:** 1 Department of Antiaging Healthcare, Changwon National University, Changwon 51140, Korea; shumy@changwon.ac.kr; 2 Department of Marine Leisure and Tourism, Youngsan University, Busan 50510, Korea; demi100@ysu.ac.kr; 3 Department of Physical Education, Changwon National University, Changwon 51140, Korea

**Keywords:** muscle strength, leg strength, balance, neurodegenerative disease, cognitive function

## Abstract

The risk of dementia increases with age. To mitigate this risk, we examined the effect of a square-stepping exercise (SSE) program on fall-related fitness and brain-derived neurotrophic factor (BDNF) levels. Twenty older adults in Korea were randomly assigned to either the experimental or control group (each group *n* = 10). Participants performed SSE for 70 min per session, twice a week, for 12 weeks with a certified instructor. The average age of the participants was 74.80 ± 6.763 years in the exercise group and 72.50 ± 6.519 years in the control group. The experiment group showed significant improvement (*p* < 0.01) in the lower muscle strength post-intervention. The paired *t*-test revealed a significant improvement (*p* < 0.01) in the experimental group and a significant difference in the interaction effect (*p* < 0.01) in the BDNF levels. There was a significant improvement (*p* < 0.05) in the BDNF levels in the experimental group and a significant decrease (*p* < 0.05) in the control group. The SSE program had a positive effect on fall-related fitness and BDNF levels.

## 1. Introduction

With the increased age of the adult population due to the advancement of modern medical technology and improvement of living conditions, neurodegenerative diseases are also on the rise. Neurodegenerative diseases are common among older adults worldwide and increase mortality [1]. The world population in 2019 was 7.71 billion, 1.3 times higher than that in 2000, and is expected to reach 10.38 billion by 2067. The proportion of the world’s population aged ≥ 65 years is expected to increase significantly, from 9.1% in 2019 to 18.6% in 2067 [2]. As aging progresses, the volume of the human brain decreases, blood flow drops, and neurochemical changes occur [3], resulting in dementia, a representative neurological disease; cognitive impairment; stroke; and other negative effects [4].

Exercise is an effective mediation to prevent and delay the decrease in cognitive functions due to aging [5]. Physical activities have positive effects on the improvement of neurotransmitter functions, such as the formation of blood vessels, synthesis of neurotransmitters, and an increase in blood flow to the brain [6]. A nine-year follow-up study of 299 people by Erickson et al. [7] reported that older adults who walked about 6–9 miles per week had greater gray matter volume in their brains compared to those who did not. The gray matter of the brain is the part that is responsible for the function of thinking, and is one of the important indicators for evaluating cognitive function. According to previous studies on the improvement of cognitive function and physical ability of older adults, cognitive physical activity programs had a positive effect on the improvement of fall prevention and cognitive function, as well as physical function such as dynamic balance of older adults [8]. After conducting the underwater psychological exercise program twice a week for 12 weeks, the cognitive function of the elderly showed a statistically significant difference compared to before exercise [9]. Zhang [10] also reported a positive effect on maintaining and improving cerebrovascular function after regular walking and band exercises for 12 weeks. Additionally, physical activity affects the neuronal derivative, increasing the manifestation of the brain-derived neurotrophic factor (BDNF) protein in specific parts of the brain [11,12] and increasing the absorption of insulin-like growth factor-1 (IGF-1) [13,14,15]. BDNF is also seen in various areas of the brain, such as the basal forebrain, hippocampus, and cortex, and enhances the level of long-term memory by stimulating the production of neurons [16], while strengthening the structural and functional changes in the brain [17]. Although BDNF levels decrease with age, production can be increased with adequate exercise and constant learning [18].

BDNF levels increase with regular exercise, and it has been reported to play an important role in increasing cranial nerve plasticity and improving cognitive function [19]. Endurance exercise increases BDNF expression and rapidly increases overall BDNF concentration in humans [20]. In particular, reduced BDNF levels in older adults are associated with hippocampal atrophy, which can lead to memory impairment [21]. Sungkarat et al. [22] reported that 6 months of Tai Chi significantly improved blood BDNF levels in patients with mild cognitive impairment (MCI). Yoon [23] reported that BDNF levels in older adults significantly improved after 12 weeks of complex exercise.

Furthermore, considering the physical functions that are important for older adults, leg imbalance and a reduction in leg strength both hinder walking ability, which leads to injuries and falls [24]. More than one in four older adults aged ≥ 65 years experience a fall every year [25,26]. Annually, approximately 50 million falls occur in older adults in Europe alone, and the medical expenditure for fall-related treatment is estimated to be 25 billion euros per year [27]. Injuries caused by falls vary widely, ranging from fractures to fatal trauma [28]. Older age, inappropriate walking patterns, and balance disorders are considered risk factors for falls [29,30]. In particular, weakening of the lower extremities has been identified as an important risk factor for falls, so leg strength exercises are very important in preventing falls [31]. Physical exercise is beneficial for older adults apprehensive about falls, balance, and their physical performance. Physical exercise can effectively improve the balance of adults aged ≥ 65 years and help prevent falls by improving posture control, and the ability to perform tasks and move safely at speed. Falling represents a health problem with a high prevalence and physical and psychological severity [32]. In addition, it may be appropriate to administer physical and cognitive training simultaneously to those at risk of falls [33]. Physical training improves mobility and cognition among older adults with subjective cognitive complaints [34,35]. A meta-analysis reported that the square-stepping exercise (SSE) is more advantageous than walking to prevent falls in older adults [33].

However, despite the positive effects of exercise being widely known, more than 60% of older adults do not exercise regularly, and the majority of older adults who suffer from dementia are unable to participate in regular exercise [36]. With many older adults facing the onset of dementia, it is essential for them to exercise regularly to prevent a loss of basic life skills, such as eating, walking, dressing, going to the bathroom, and taking a shower. Older adults’ participation in exercise is correlated with subjective health status [37]. In addition, social activities are reduced for various reasons such as economic power, meaningful others, depression, physical decline, or chronic disease [38,39,40]. Therefore, it is considered that follow-up studies, including this improvement of relationship, should be conducted in studies targeting the older adults.

SSE is a form of walking exercise that can be easily performed as a group and at low cost because it does not require expensive equipment. It was developed to allow older adults to exercise indoors at their convenience and without the challenges, such as rain or severe cold, they may face when walking outdoors. Moreover, SSE includes both physical and cognitive exercises, and because it is performed as a group, it can potentially promote social interaction [41].

SSE involves forward, backward, left, right, and diagonal movements using a mat divided into 25 cm squares on a surface 100 cm wide and 250 cm long. There are 196 movements, which are categorized into beginner 1, beginner 2, intermediate 1, intermediate 2, intermediate 3, high 1, high 2, and high 3 movements, depending on the difficulty of the pattern, which can motivate and challenge the individual. The intensity of exercise increases according to the difficulty of the pattern (Square Stepping Association, 2011).

In a study conducted on 41 older adults (21 in the exercising group and 20 in the control group) aged > 60 years, cognitive functions were enhanced in the exercise group that performed 40-min SSE sessions three times a week over 16 weeks [42]. In addition, in a study of 68 adults aged > 65 years in Japan, significant improvements in lower extremity strength, balance, and agility were observed after conducting SSE twice a week for 12 weeks [43]. According to Lee and Kim [44], although not significantly, the cognitive function scores of older adults with MCI improved after performing SSE for 12 weeks. In a study including not only older but also young adults, the modified card sorting task score, which measures abstract reasoning, mental flexibility, and problem-solving ability, was significantly improved after SSE [45]. Thus, the positive effects of SSE have been proven in multiple reports. Regular exercise by older adults is believed to not only effectively enhance their fitness but also maintain and improve their cognitive functions and slow the onset of dementia by increasing secretion of neurotrophic factors, such as BDNF and IGF-1. Therefore, to improve the assessment of the preventive aspect of fall-related fitness and cognitive degeneration, studies should be conducted among older adults without neurological diseases, and measurements should be performed multilaterally. These research results can provide baseline data to improve research and develop solutions for the maintenance of quality of life in the later years for older adults.

There are no reports in the literature on the effect of SSE on nerve derivatives and increased BDNF and IGF-1 expression. Therefore, in this study, the effect of SSE on fall-related physical strength and BDNF and IGF-1 levels in older adults was investigated.

## 2. Materials and Methods

### 2.1. Participant Selection

Participants were recruited from a local community. However, it was difficult to recruit a large number of participants because SSE is not yet a generalized exercise program in Korea. The criteria for selecting participants were as follows: age 65–90 years, lack of regular exercise (twice per week for 1 week), willingness to accurately understand the study purpose and procedure, and voluntary participation. Of the 38 people who were initially recruited, those diagnosed with dementia, depression, severe cardiovascular disease, or mental illness were excluded. In addition, the finally included participants did not have confirmed dementia and had a total score of 20 or more in the Korean Version of Mini-Mental State Examination (K-MMSE).

Participants were randomly assigned to the experimental group (*n* = 10) and control group (*n* = 10) using a computer-generated permutation block randomization design.

The study was approved by the ethical board at Changwon National University in Korea (approval number: 1040271-201811-HR-031). The study was explained in detail to the participants, and a written informed consent was obtained from all participants. The participants were also provided with the option to withdraw from the study at any time.

### 2.2. Exercise Methodology

**Figure 1:**
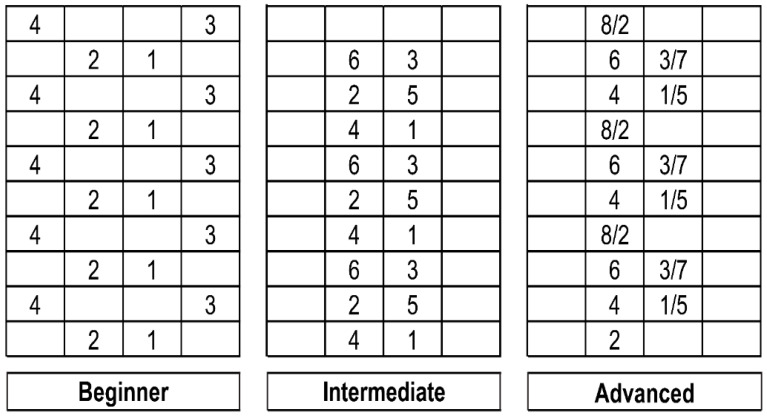
Examples of square-stepping exercise patterns.

SSE sessions of 70 min each were conducted twice a week over a 12-week period. In every class, the participants were asked not to engage in sports other than SSE. Subjects who participated in other regular exercise programs related to cognitive function and fall-prevention fitness during the study period or did not participate in more than 4 of the 24 sessions were excluded from the analysis. In addition, if the participant experienced a change in face color or dizziness during the exercise, the session stopped immediately. All participants included in this study attended the sessions at least 21 times. The warm-up for the exercise involved walking and stretching; the cool-down comprised 10 min of stretching, and the main exercise comprised a 50-min program. First, a beginner level 1 program was conducted repeatedly to promote confidence and self-efficacy; once the participants were comfortable with the entire beginner level 1 pattern, they proceeded to the beginner level 2 program, and the beginner level 1 program was performed as a warm-up for the main exercise, as shown in Figure 1.

The control group was asked to limit regular physical activity other than daily life activities through phone calls twice a month. Both the experimental and control groups were asked to maintain their normal lifestyle during the study period and to inform the research manager of sudden health problems or changes in their daily life. In addition, participants could communicate and ask questions at any time. Free education for dementia prevention and fall prevention were provided to the control group in this study. Finally, a gift was provided. The implementation method of SSE is shown in Table 1.

### 2.3. Fall-Related Fitness and Blood Measurement

To evaluate athletic ability, two factors were measured: the muscle strength of the extremities, and balance related to fall-related physical strength. For comparison, these factors were measured twice, before the start of the program and immediately after the end of the program. Muscle strength of the lower extremities was measured by asking the participant to alternatively stand and sit in a chair for 30 s. The subjects were asked to place their arms on their chest in an X-shape while they sat on a stationary chair with a vertical backrest and the soles of their feet resting on the floor. A start signal was given when the patient was in position, and the patient was asked to fully stand up and sit on the chair again to achieve accurate posture. A researcher first demonstrated the process to the participants before starting the experiment. A stopwatch was used, and the number of repetitions with accurate posture over 30 s was recorded. For balance measurement, a one-legged closed-eye test was conducted and timed using a stopwatch. The participants stretched both arms to their sides as the preparatory posture. Then, they slowly raised one foot when the researcher said “start”, with their eyes closed. They were then asked to maintain this posture. The time until excessive movement or placement of the leg back on the floor was measured in seconds up to the second decimal place. This test was performed twice.

**Figure 2:**
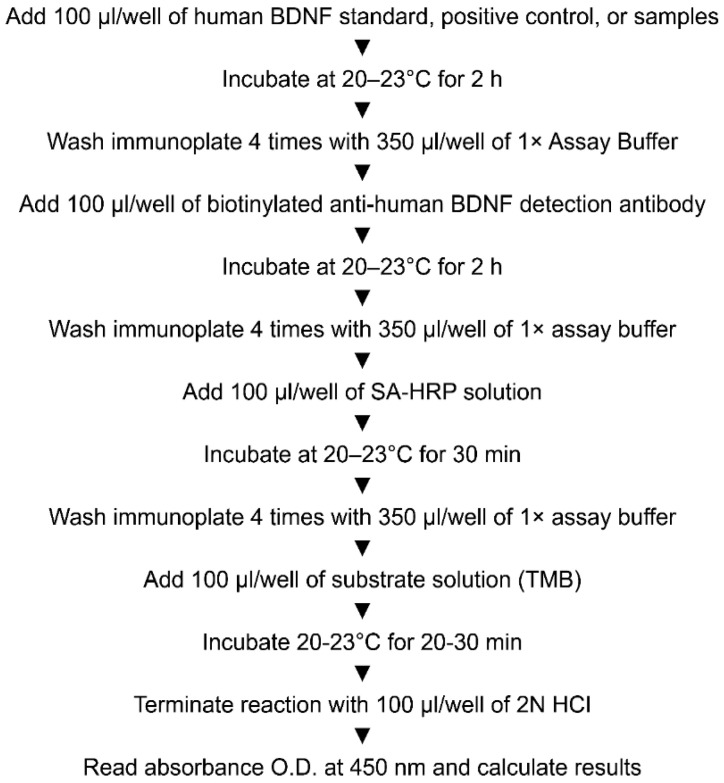
Summary of assay protocol. BDNF, brain-derived neurotrophic factor.

As shown in Figure 2, blood samples were collected between 9 am and 10 am. Similar to the evaluation of the exercise ability, blood samples were obtained before and after the start of the program. The nurse collected the blood samples according to the doctor’s instructions. The participants were asked to maintain an empty stomach for at least 12 h before blood collection to minimize the effect of diet. Exercise within 48 h before the test was prohibited to minimize the effect of exercise. The BDNF levels were assessed using the standardized enzyme-linked immunosorbent assay (ELISA) test method from clinical trials, and the specific protocol is presented in Figure 2. IGF-1 was stored at 15–25 °C for approximately 15 min to coagulate blood in the sample container after blood collection, serum was obtained using a centrifuge (3000 RPM × 15 T), and analysis was conducted on the same day.

### 2.4. Statistical Analysis

SPSS (IBM SPSS Statistical for Windows, version 25.0, IBM Corp., Armonk, NY, USA) software was used to perform statistical analysis. The average and standard deviations of each group were calculated and mapped to verify the difference in the age of each group. A two-way ANOVA with repeated measures was performed for the interaction effects. Paired *t*-tests and independent *t*-tests were conducted to validate significant interactions for the group and the group × time. Cohen’s D for the group effect size was calculated and classified into small (smaller than 0.2), medium (0.21–0.79), and large (larger than 0.8). It showed effect size with a confidence interval of 95% (BDNF = 0.75, IGF 1 = 0.43, muscle strength of lower extremities = 0.79, balance = 0.57). The significance level (α) of all the hypotheses was set at α = 0.05.

## 3. Results

The study was conducted between April and June 2019. Of the 38 participants, those whose participation rate was less than 80% or who resigned during the investigation owing to personal circumstances, such as eye disease and hospitalization, were excluded. The final number of participants was 10 each in the experimental and control groups. The average age of the participants was 74.80 ± 6.763 years in the exercise group and 72.50 ± 6.519 years in the control group. No falls or injuries related to the study were reported. The demographic characteristics of the experimental and control groups are shown in Table 2. The results of each group after completing SSE for 12 weeks are shown in Table 3, Table 4 and Table 5.

### 3.1. Fall-Related Fitness

#### 3.1.1. Muscle Strength of Extremities

In the experimental group, the pre- and post-test values for the muscle strength test were 22.20 ± 6.97 and 25.80 ± 9.02 repetitions, respectively; in the control group, the pre- and post-test values were 18.80 ± 5.20 and 17.40 ± 7.06 repetitions, respectively. Repeated measurement two-variant analysis was conducted to verify the change in the lower-limb muscle strength according to the measurement timing between each group. The paired *t*-test results to verify the interaction effect showed that the experimental group improved after exercise (*p* < 0.01), and there was no difference in the control group (*p* > 0.05). An independent sample *t*-test was performed to determine whether there was a difference between the groups. Consequently, the pre-test (t = 1.236, *p* = 0.232) and post-test (t = 2.320, *p* = 0.032) results showed no difference between the groups in the pre-test, although there was a significant difference between the groups in the post-test.

#### 3.1.2. Balance

In the experimental group, the pre-test mean value was measured at 9.00 ± 9.89 s, whereas the post-test mean value was 15.13 ± 9.57 s. In the control group, the pre-test mean value was 11.12 ± 13.42 s, whereas the post-test value was 6.93 ± 7.09 s. According to the repeated measurement two-way variance analysis, there was no significant difference between the two groups (*p* > 0.05), whereas there was a significant difference in the point of time for the group and interaction effects (*p* < 0.05) for balance. According to the independent sample *t*-test for the pre-test (t = −0.401, *p* = 0.693) and post-test (t = 2.179, *p* = 0.043) values, there was no difference in the pre-test values of the two groups, although there was a significant difference between their post-test values.

#### 3.1.3. BDNF

In the experimental group, the pre-test and post-test BDNF values were 1393.00 ± 208.88 pg/mL and 1492.23 ± 256.59 pg/mL, respectively. In the control group, pre-test and post-test BDNF values were 1303.61 ± 289.28 pg/mL and 1181.28 ± 279.43 pg/mL, respectively

Repeated measurement two-way ANOVA showed no significant difference between the two groups (*p* > 0.05) at the point of time within a group for BDNF levels. In addition, there were no significant differences in the interaction effects (*p* < 0.01). Thus, there was no significant difference in either the post hoc test results between the two groups, or in the point of time within a group (*p* > 0.05) for BDNF; however, the paired *t*-test showed a significant increase in BDNF values post-exercise compared with pre-exercise in the experimental group (*p* < 0.01). Notably, there was a significant difference between the control groups (*p* < 0.05). As a result of the independent sample *t*-test for the pre-test (t = 0.792, *p* = 0.439) and post-test (t = 2.592, *p* = 0.018), there was no difference between groups in the pre-test, although there was a significant difference between the groups in the post-test.

#### 3.1.4. IGF-1

In the experimental group, the pre-test and post-test IGF-1 values were 147.45 ± 42.87 ng/mL and 140.06 ± 50.58 ng/mL, respectively. In the control group, the pre-test and post-test IGF-1 values were 144.67 ± 39.87 ng/mL and 109.86 ± 18.56 ng/mL, respectively.

Repeated measurement two-way variance analysis revealed no significant difference between the two groups (*p* > 0.05), whereas there was a significant difference in the time point between the two groups (*p* < 0.01). In addition, there was no significant difference in the post hoc test results between the two groups and point of time within a group (*p* > 0.05). There was also no significant difference in the paired *t*-test for balance (*p* < 0.05). According to the independent sample *t*-test for the pre-test (t = 0.150 and *p* = 0.882) and post-test (t = 1.772 and *p* = 0.093), there was no difference between the groups in either test.

## 4. Discussion

This study investigated the effects of older adult fall-related fitness and BDNF levels through participation in a 12-week SSE program. There was no significant difference in the leg strength in the control group, whereas there was a significant improvement in the experimental group. In addition, BDNF levels were significantly increased in the experimental group but significantly decreased in the control group. The results of this study suggest that SSE can improve fall-related physical strength and positively affect brain nerve growth factor levels.

Decreased muscle strength in the lower extremities of older adults increases the risk of falls [46,47]. Previous studies have shown that lower extremity muscle strength can be maintained or improved through physical activity. Kim et al. [48] reported that lower extremity muscle strength improved after a fall prevention exercise program, and Um et al. [49] reported that older adults had improved lower extremity strength after 12 weeks of resistance exercise. There was significant improvement in the muscle strength of the extremities of the experimental group in this study. SSE reinforces the muscle strength of the extremities by applying a load to the legs, requiring individuals to not only remember and follow a pattern, but to also use the muscle strength of the extremities that are usually unused, such as lifting one leg and maintaining a position or moving forward using the heel and toe. Performing SSE for 12 weeks enhances muscle strength in older adults.

Compared with their younger counterparts, older adults have lower walking stability in response to new, small environmental changes [50]. In recent years, many studies have proposed applying balance competency training to manage and improve the balance of older adults [51]. Lee [52] reported that the balance ability of obese older adult women improved after coordinated locomotor training, and Kim and Heo [53] reported the improved balance ability of older adults after balance exercises using Swiss balls. In this study, the change in balance was not significantly different between the experimental and control groups. However, in the experimental group, balance nominally increased post-intervention, implying that SSE had a positive effect on balance. Additionally, the control group showed a sharp decrease in the post-test. This can be seen in a similar context to the study by Cho [54], who reported a sharp rise in equilibrium in the exercise group: the equilibrium (one-leg balance closed-eye test) increased from 18.09 ± 6.98 to 29.18 ± 9.87 in the group of older adults (61 to 65 years old) who performed isokinetic exercise (median speed). The sensory systems that contribute to the equilibrium include the visual, somatosensory, and vestibular systems. As we age, our proprioceptive senses become dull, our cognitive abilities and our sense of vibration are reduced, our weight transfer time is longer, and our reaction time to any situation is delayed [55]. In particular, the visual system provides information, including the shape or state of the environment, distance, and ground conditions in dangerous situations, and induces changes in posture by providing the required intensity of movement and degree of the current body position [56]. As such, a wide variety of factors affect older adult balance; for example, standing on one leg is three times more likely to cause a fall [57]. However, the balance test in this study required balance maintenance while standing on one leg with eyes closed. Therefore, it is thought that minor changes in each individual over 12 weeks had a very large effect on the post-test results. In addition, the participants in this study were older, with an average age of 74.80 ± 6.763 and 72.50 ± 6.519 years in the experimental and control groups, respectively, and the difference in the pre- and post-test results was greater.

BDNF is abundant in the brain, and it is a neurotropine with multiple functions and an important role in brain cell development, maintenance, and plasticity [58]. Expression of BDNF decreases with aging, which may explain the decline in the cognitive functions of the elderly [59]. A study of 2131 elderly people without dementia showed that the higher the serum BDNF level, the lower the incidence of dementia [60]. In addition, it was reported that increased BDNF levels through exercise improved the cognitive function of mice with Alzheimer’s [61].

As shown in previous studies, physical activity is related to an improvement in blood BDNF concentration; the blood BDNF levels increase following both long-term and single-time exercise [62]. In this study, we found that SSE was effective in improving blood BDNF levels in older adults. This improvement is believed to have stimulated brain cells by simultaneously performing pattern memory tasks for each level of difficulty. This finding is consistent with those of other studies. Vaughan et al. [63] reported that, following a combined exercise program for older adult women twice a week over 16 weeks, BDNF levels increased significantly, and because of this, the speed of speech, ability to concentrate, and fluency also increased [63]. Choi et al. [64] reported that, after conducting combined exercise in older adult women for over 12 weeks, BDNF levels also increased noticeably. Similar results have been reported in studies using mice. Ki et al. [65] reported a significant increase in BDNF mRNA expression in the hippocampus after 16 weeks of treadmill exercise with ovarian-resected mice, and Jo et al. [66] reported a significant increase in BDNF expression in the hippocampus after 12 weeks of swimming.

Conversely, Maass et al. [67] claimed that, after conducting exercise for over 3 months in 40 healthy older adults divided into a running machine training group and progressive muscle relief and stretching group, although the health levels of the participating older adults increased significantly, there was no effect on the blood levels of BDNF and IGF-I. Voss et al. [68] claimed that, after conducting aerobic exercise (walking) for 1 year in healthy older adults, there was no significant effect on the serum levels of BDNF and IGF-I compared with the control group that performed muscle stretching [68,69,70].

In long-term exercise, the reason BDNF does not increase during stabilization is the enhanced efficient removal rate; the circulating BDNF protein tends to move to damaged tissue first to be used during stabilization [71]. Moreover, the BDNF in the blood changes in form over time [5,72], and the manifestation period of increased BDNF levels through exercise over long periods of time are reportedly proportionate to the quantity and period of exercise [73,74]. Despite reports claiming that it is difficult to enhance the blood BDNF levels, we found that they significantly improved in older adults above an average age of 74 years.

To date, there have been no studies evaluating the change in BDNF levels following an SSE program among older adults. Therefore, although a detailed comparison is difficult, significantly elevating the BDNF in the blood of older adults above the average age of 74 for a 12-month period, as proposed above, suggests that SSE has an extremely positive effect on BDNF related to learning and memory in older adults.

IGF pathways play an essential role in brain development, including angiogenesis and neurogenesis [75]. Potentially, the higher the concentration of IGF-I, the more protective it is against neurodegeneration [76]. In addition, the IGF-I concentration is positively associated with the cognitive state [77].

IGF-1 is an important growth factor that regulates synaptic plasticity, density, and neurotransmission, and is involved in vascular maintenance and remodeling. In addition, a decrease in IGF-1 levels due to aging is associated with decreased brain function [78,79,80,81,82,83,84]. IGF-1 plays an important role in improving serum BDNF by converting proBDNF into mBDNF in the central nervous system; the increase in post-exercise IGF-1 levels explains the increase in the post-exercise BDNF levels [85].

As for the change in the IGF-1 levels in this study, there was no significant difference within the experimental group before and after the intervention, although there was a significant decrease in the control group. Arazi et al. [78] reported a significant increase in serum IGF-1 levels in older adult men after muscle strengthening and endurance exercises compared with those before exercise. Han [86] stated that conducting underwater, coordination, and aerobic exercises in older adult women over 12 weeks had no effect upon their IGF-1 levels in any of the three groups. Additionally, Lee [87] reported no effect on IGF-1 after conducting walking, yoga, and underwater exercises for 12 weeks in older adult women. The increase in IGF-1 levels after conducting regular exercise has been related to the age of the research participants as well; however, Hakkinen et al. [88] claimed that IGF-1 improved more in a group of 30-year-olds compared with 60-year-olds after exercising for 10 weeks. As such, IGF-1 tends to decrease during the aging process [89]. However, although it decreased significantly in the control group in this study, there was no significant difference in the experimental group. These results demonstrate that exercise might help maintain the IGF-levels in older adults. According to a report by Berg and Bang [90], the increase in IGF-1 occurs only during the training period and immediately drops within 10 min of training; thus, there is a possibility that IGF-1 levels fluctuate quickly during and after training, which has a temporary effect on the cerebrovascular structure of the hippocampus and neural functions.

Therefore, owing to the sensitivity of the tests, such as the IGF-1 blood test, to several factors, it is necessary to develop a method to control the participants’ daily routine in the future, although this seems restrictive. Furthermore, although the effects of short- and long-term exercise on fall-related fitness, cognitive function, and BDNF levels in many studies are inconsistent, there is a need for studies that have been conducted using various protocols.

This study has some limitations. First, the psychological factors of the participants were not controlled. Although there were many interactions between the researchers and patients, perfect control of the psychological aspect would be difficult. Second, the participants’ daily activities were not controlled. Third, the sample size was small; therefore, the results may differ from those of large-scale clinical studies, and the results of this study should be cautiously interpreted. To compensate for these limitations, large-scale studies involving long-term follow-up with multiple researchers must be conducted.

## 5. Conclusions

The present study showed that SSE had a positive effect on fall-related fitness and BDNF levels. SSE enhances fall-related fitness through repetitive and continuous steps, changing of direction, and accurately placing the foot in a 25 cm × 25 cm square. It also has a positive effect on the BDNF levels because the patient is required to remember the pattern, which stimulates the brain cells. In addition, if older adults with decreased muscle strength due to aging and non-usage engage in SSE regularly, their fitness and blood flow to the brain would improve, and it would be possible to maintain and improve the function of the brain blood vessels, as well as the cognitive functions. Based on these results, exercise programs that break away from simply following movements while stimulating the individual to think and act continuously are more meaningful for older adults. Accordingly, the SSE program can be applied not only to older adults, but also to various classes and groups in future research.

## Figures and Tables

**Table 1:** Square-stepping exercise implementation method.

	Section	Contents	Time
	Warm-up	Walking and stretching	10 min
1 week	Main exercise	Beginner level 1	50 min
2–3 weeks		Beginner level 1	25 min
		Beginner level 2	25 min
4–12 weeks		Beginner level 1~2	10 min
		Review	10 min
		New pattern (intermediate level)	30 min
	Cool-down	Stretching	10 min

**Table 2:** Participant characteristics.

Characteristics	Exercise Group (*n* = 10)	Control Group (*n* = 10)
Age (years), mean ± SD	74.80 ± 6.763	72.50 ± 6.519
Height (cm)	158.12 ± 7.724	160.39 ± 6.327
Weight (kg)	58.10 ± 7.214	59.80 ± 8.220
Sex (M:F)	3:7	3:7
Fall experience (yes:no)	2:8	5:5
MMSE (range 0–30)	26.10 ± 2.885	26.20 ± 3.327

Note: SD, standard deviation; M, male; F, female; MMSE, Mini-Mental State Examination.

**Table 3:** Results of two-way ANOVA.

Item		Source	SS	DF	MS	F-Value	*p*
Muscle strength of lower extremities	Between						
		Group (A)	348.100	1	348.100	3.542	0.076
		Error	1768.800	18	98.267		
	Within						
		Time (B)	12.100	1	12.100	2.357	0.142
		A × B	62.500	1	62.500	12.175	0.003 **
		Error	92.400	18	5.133		
Balance	Between						
		Group (A)	92.598	1	92.598	0.530	0.476
		Error	3142.287	18	174.571		
	Within						
		Time (B)	9.428	1	9.428	7.548	0.013 *
		A × B	266.153	1	266.153	7.548	0.013 *
		Error	634.709	18	35.262		
BDNF	Between						
		Group (A)	400678.287	1	400678.287	3.142	0.093
		Error	2295244.645	18	127513.591		
	Within						
		Time (B)	1333.217	1	1333.217	0.165	0.690
		A × B	122723.192	1	122723.192	15.148	0.001 **
		Error	145829.277	18	8101.627		
IGF-1	Between						
		Group (A)	2719.201	1	2719.201	1.000	0.331
		Error	48952.245	18	2719.569		
	Within						
		Time (B)	4452.100	1	4452.100	9.989	0.005 **
		A × B	1879.641	1	1879.641	4.217	0.055
		Error	8022.629	18	445.702		

Note: * *p* < 0.05; ** *p* < 0.01; ANOVA, analysis of variance; SS, sum of square; DF, degrees of freedom; MS, mean square; BDNF, brain-derived neurotrophic factor; IGF-1, insulin-like growth factor.

**Table 4:** Paired *t*-test results.

Item	Group	N	Time of Measurement		DF	t	*p*
			Pre	Post			
Muscle strength of lower extremities (repetitions)	Exercise	10	22.20 ± 6.97	25.80 ± 9.02	9	−4.070	0.003 **
	Control	10	18.80 ± 5.20	17.40 ± 7.06	9	1.242	0.246
Balance(s)	Exercise	10	9.00 ± 9.89	15.13 ± 9.57	9	−2.034	0.072
	Control	10	11.12 ± 13.42	6.93 ± 7.09	9	1.868	0.095
BDNF (pg/mL)	Exercise	10	1393.00 ± 208.88	1492.23 ± 256.59	9	−2.312	0.046 *
	Control	10	1303.61 ± 289.28	1181.28 ± 279.43	9	3.271	0.010 *
IGF-1 (ng/mL)	Exercise	10	147.45 ± 42.87	140.06 ± 50.58	9	0.952	0.366
	Control	10	144.67 ± 39.87	109.86 ± 18.56	9	3.205	0.011 *

Note: * *p* < 0.05; ** *p* < 0.01; BDNF, brain-derived neurotrophic factor; IGF-1, insulin-like growth factor

**Table 5:** Independent *t*-test results.

Item	Time of Measurement	Group		t	*p*
		Exercise (*n* = 10)	Control (*n* = 10)		
Muscle strength of lower extremities (repetitions)	Pre	22.20 ± 6.97	18.80 ± 5.20	1.236	0.232
	Post	25.80 ± 9.02	17.40 ± 7.06	2.320	0.032 *
Balance(s)	Pre	9.00 ± 9.89	11.12 ± 13.42	−0.401	0.693
	Post	15.13 ± 9.57	6.93 ± 7.09	2.179	0.043 *
BDNF (pg/mL)	Pre	1393.00 ± 208.88	1303.61 ± 289.28	0.792	0.439
	Post	1492.23 ± 256.59	1181.28 ± 279.43	2.592	0.018 *
IGF-1 (ng/mL)	Pre	147.45 ± 42.87	144.67 ± 39.87	0.150	0.882
	Post	140.06 ± 50.58	109.86 ± 18.56	1.772	0.093

Note: * *p* < 0.05; BDNF, brain-derived neurotrophic factor; IGF-1, insulin-like growth factor.

## Data Availability

Not applicable.
